# Comparative analysis of deeply phenotyped GBM cohorts of ‘short-term’ and ‘long-term’ survivors

**DOI:** 10.1007/s11060-023-04341-3

**Published:** 2023-05-26

**Authors:** Archita Biswas, Manuela Salvucci, Kate Connor, Heiko Düssmann, Steven Carberry, Michael Fichtner, Ellen King, Brona Murphy, Alice C. O’Farrell, Jane Cryan, Alan Beausang, Josephine Heffernan, Mattia Cremona, Bryan T. Hennessy, James Clerkin, Kieron J. Sweeney, Steve MacNally, Francesca Brett, Philip O’Halloran, Orna Bacon, Simon Furney, Maite Verreault, Emie Quissac, Franck Bielle, Mohammed H. Ahmed, Ahmed Idbaih, Sieger Leenstra, Ioannis Ntafoulis, Federica Fabro, Martine Lamfers, Anna Golebiewska, Frank Hertel, Simone P. Niclou, Romain Tching Chi Yen, Andreas Kremer, Gonca Dilcan, Francesca Lodi, Ingrid Arijs, Diether Lambrechts, Manasa Kalya Purushothama, Alexander Kel, Annette T. Byrne, Jochen H.M. Prehn

**Affiliations:** 1 Department of Physiology and Medical Physics, Centre for Systems Medicine, Royal College of Surgeons in Ireland, 123 St Stephen’s Green, Dublin 2, Dublin, D02 YN77 Ireland; 2 Department of Neuropathology, Beaumont Hospital, Dublin 9, Dublin, Ireland; 3 Department of Medicine, Royal College of Surgeons in Ireland and Beaumont Hospital, Dublin 9, Dublin, Ireland; 4 Department of Neurosurgery, Beaumont Hospital, Dublin 9, Dublin, Ireland; 5 DMU Neurosciences, Service de Neurologie 2-Mazarin, Sorbonne Université, AP-HP, Institut du Cerveau - Paris Brain Institute - ICM, CNRS, Hôpitaux Universitaires La Pitié Salpêtrière - Charles Foix, Inserm, F-75013 Paris, France; 6 Dept of Neurosurgery Brain Tumor Center, Erasmus University Medical Center, Wytemaweg 80, 3015 CN Rotterdam, The Netherlands; 7 NORLUX Neuro-Oncology laboratory, Department of Cancer Research, Luxembourg Institute of Health, 6A, Rue Nicolas-Ernest Barblé, L-1210 Luxembourg, Luxembourg; 8 Information Technology for Translational Medicine, 27, Rue Henri Koch - House of BioHealth, L-4354 Esch-sur-Alzette, Luxembourg; 9 VIB-KU Leuven Cancer for Cancer Biology, Onderwijs en Navorsing 5, Herestraat, 49, 3000 Leuven, Belgium; 10 geneXplain GmbH, Am Exer 19b, 38302 Wolfenbüttel, Germany; 11 Faculty of Sciences, Technology and Medicine, University of Luxembourg, L-4365 Esch-sur-Alzette, Luxembourg

**Keywords:** Glioblastoma, Reverse phase protein array, RNA-sequencing, Transcriptomics, Cilium, Cell cycle, Apoptosis, Short term survivors, Long term survivors

## Abstract

**Background:**

Glioblastoma (GBM) is an aggressive brain cancer that typically results in death in the first 15 months after diagnosis. There have been limited advances in finding new treatments for GBM. In this study, we investigated molecular differences between patients with extremely short (≤ 9 months, Short term survivors, STS) and long survival (≥ 36 months, Long term survivors, LTS).

**Methods:**

Patients were selected from an in-house cohort (GLIOTRAIN-cohort), using defined inclusion criteria (Karnofsky score > 70; age < 70 years old; Stupp protocol as first line treatment, IDH wild type), and a multi-omic analysis of LTS and STS GBM samples was performed.

**Results:**

Transcriptomic analysis of tumour samples identified cilium gene signatures as enriched in LTS. Moreover, Immunohistochemical analysis confirmed the presence of cilia in the tumours of LTS. Notably, reverse phase protein array analysis (RPPA) demonstrated increased phosphorylated GAB1 (Y627), SRC (Y527), BCL2 (S70) and RAF (S338) protein expression in STS compared to LTS. Next, we identified 25 unique master regulators (MR) and 13 transcription factors (TFs) belonging to ontologies of integrin signalling and cell cycle to be upregulated in STS.

**Conclusion:**

Overall, comparison of STS and LTS GBM patients, identifies novel biomarkers and potential actionable therapeutic targets for the management of GBM.

**Graphical abstract:**

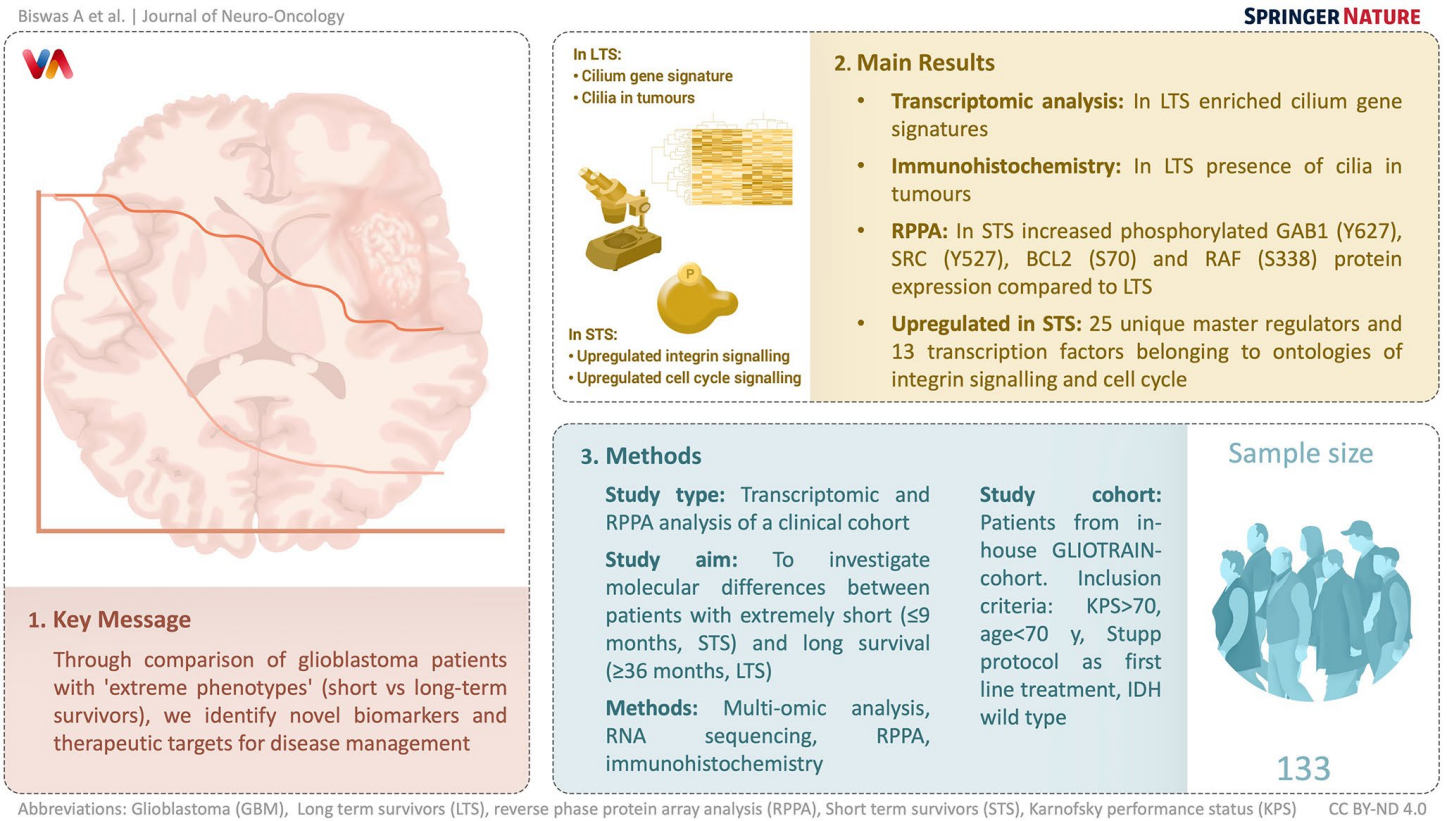

**Supplementary Information:**

The online version contains supplementary material available at 10.1007/s11060-023-04341-3.

## Introduction

Glioblastoma (GBM) is the most aggressive primary glioma in adults [1], despite improvements in the standard of care (Stupp protocol), surgical advances and targeted therapies, patient outcome remains poor [2, 3]. Indeed, as almost all GBM patients suffer from disease progression and recurrence, there is an urgent need to identify new treatments for GBM. Notably, few biomarkers are currently available for prognostication in the GBM setting. The most robust prognostic molecular biomarker is MGMT promoter methylation [4]. Detection of IDH mutation associated with better in diffuse glioma rules out the diagnosis of GBM according to the novel classification published by the World Health Organisation (WHO) [5]. While these molecular biomarkers harbour value for the clinical management of GBM patients, and may predict response to therapy, MGMT promotor methylation is not exhaustive and, in many cases, fail to accurately predict the patient outcome or therapeutic response [6]. Therefore, there exists an urgent need to identify and develop effective biomarkers associated with prognosis and response to treatment, particularly for newer treatment modalities.

In addition to the aforementioned biological markers, Karnofsky Performance status (KPS) and extent of resection (EoR) can be indicative of patient outcome [7]. Interestingly, a small number of patients (as little as 2%) respond well to standard of care (SOC) therapy and survive beyond 36 months. This unique patient cohort are defined as long-term survivors (LTS) [8]. Previous efforts to define the unique LTS population via analysis of clinical, genetic, epigenetic and molecular feature patterns has been unsuccessful, and no robust biomarkers or signatures have been identified. Therefore, the clinical management of GBM, and in particular the identification of LTS GBM patients, urgently requires novel and comprehensive biomarkers.

In this study, we aimed to investigate the differences in molecular characteristics and biological pathways in GBM tumors from LTS when compared with short-term survivors (STS patients). We hypothesized that based on the concept of ‘natural pre-selection’, such ‘extreme phenotypes’ can be compared to identify and characterise phenotypes and signaling pathways that are functional drivers of GBM progression (activated in STS) or resistance (activated in LTS). These could represent novel therapeutics targets as well as novel biomarkers. This strategy has previously been successful in elucidating metabolic aberrations leading to obesity, but so far has not been widely applied to GBM [9]. We therefore selected STS and LTS patients from an in-house cohort (GLIOTRAIN; GT) [10], and performed an unbiased, in-depth transcriptomic and (phospho)proteomic analyses on the STSs and LTSs of this cohort. Combined transcriptomic and (phospho)proteomic analyses revealed putative biomarkers which may be prognostic and facilitate the discovery of new treatments for GBM.

## Materials and methodology

### Clinical data collection for GLIOTRAIN (GT) patient cohort

The GT cohort comprises GBM tumors collected across four clinical centers: RCSI (Beaumont Hospital, Dublin, Ireland), ICM (Paris, France), EMC (Rotterdam, Netherlands), and LIH (Luxembourg, in collaboration with the Neurosurgical Department of the Centre Hospitalier de Luxembourg). All patient samples met the GLIOTRAIN biobank inclusion criteria [10] and were divided into Short-term survivors (STS) (≤ 9 months), Intermediate-term survivors (ITS) (> 9 and < 36 months), and Long-term survivors (LTS) (≥ 36 months) based on their overall survival (OS) among the N = 133 included patients.

### Reverse phase protein array (RPPA) analysis

The entire GT cohort, consisting of N =133 samples, were analysed via RPPA [11]. 7 samples were removed due to initial quality control fail. 72 antibodies were selected to quantify proteins associated with different signalling pathways [12]. Microvigene software (v5.1) was used to generate spot signal intensities and normalize the spots via protein loading [13]. The data was divided into linear values by the correction factor to obtain the normalized value [13]. Final protein count data was normalized using z-score technique. ConsensusClusterPlus package (v1.48.0) was implemented for clustering analysis with specific parameters; innerLinkage = “average”, finalLinkage = “average” and distance= “spearman”.

### RNA-seq data and transcriptomics analysis

The GT RNA-seq transcriptomics dataset was downloaded from the curated GT database (tranSMART) [10]. Count data were normalized using variance stabilizing transformation (VST). DESeq2 (v3.11) package was used to identify the differentially expressed genes (DGEs) from RNA-Seq data. Heatmaps were generated using ‘Complex Heatmap’ (v1.0.12) and Gene Ontology (GO), and Volcano plots generated using Bioconductor packages ‘clusterProfiler’ (v3.11) and ‘EnhancedVolcano’ (v3.11). GBM subtype classification [Classical (CL), Mesenchymal (Mes), Proneural (PL)] was performed using the ‘gliovis’ tool (http://gliovis.bioinfo.cnio.es/) as previously published [10].

### Master regulator analysis

To obtain master regulators, the Genome Enhancer pipeline was employed (www.geneXplain.com) [14–16]. Significantly upregulated genes in STSs were analsyed using Composite Module Analyst (CMA) [17] to detect potential enhancers. To identify the important transcription factors (TFs) we:

- ranked TF motifs (PWMs) based on a Yes/No ratio of their frequency in promoter sequences. A set of promoter sequences of interested genes is called the Yes set, while the promoter sequences of unchanged genes under the same experimental condition are called the No set. Motifs with a high Yes/No ratio and statistically significant enrichment of occurrences in Yes sequences, as determined by the binomial p-value, were considered important.
- We computed a regulatory score that measures the TF’s involvement in controlling genes that encode master regulators. The TRANSPATH® database and graph search algorithms were used to identify common regulators of the revealed TFs [18]. Master regulators were ranked using logFC, CMA score (indicating the gene’s potential to be regulated by TFs of interest), and master regulator score (indicating the gene product’s potential to regulate the activity of TFs).

### Immunostaining

Immunostaining was performed as previously published [19]. Slides were deparaffinized using xylene, EthOH/water gradient (100%, 90%, 70%, 50%, 30%). Antigen retrieval was applied using a microwave (400 W for 25 min). Slides were blocked using a diluted blocking buffer (5% BSA, 5% goat serum in PBS with 0.2% triton-x) and incubated (100 min/RT) with anti-acetyl-alpha Tubulin primary antibody (clone 6-11B-1, Merck, Sigma Aldrich), followed by AlexaFluor-594 donkey anti-mouse secondary antibody (1:1000 for 90 min/RT, Life Technologies). Finally, slides were mounted with DAPI mounting medium and fluorescence signal acquired using a Nikon TE 300 Fluorescence Microscope and a SPOT RT SE 6 CCD Camera. Appropriate filter blocks for DAPI or AlexaFluor 594 were used to capture images with a Nikon 60 × 1.4 NA oil immersion objective.

### Statistical analysis

The analysis used R (v4.0-4.0) with the functions ‘pairwise’ and ‘likelihood t test’ for univariate Cox regression models in RPPA and RNA-seq transcriptomic analysis. For RPPA analysis, protein level differences between clusters were identified using one-way ANOVA with Tukey’s post hoc tests. Univariate survival analysis was performed using the ‘survminer’ (v0.4.4) and ‘survival’ (v2.44-1.1) packages. Visualization of all plots was done using ‘ggpubr’ (v0.2) and ‘ggplot2’ (v3.1.1).

## Results

### Acquisition and stratification of short- and long-term GBM survivors

To establish a cohort of primary GBM samples for downstream interrogation, GBM tumour samples (N = 128) were procured from the GLIOTRAIN (GT) biobank [10]. An additional N = 5 samples from the LIH biobank were subsequently added to the GT cohort to form the expanded GT-cohort (N = 133 patients samples total). Within this expanded GT-cohort, patients were stratified based on OS, identifying N = 18 STS, N = 82 Intermediate Term Survivors (ITS), and N = 33 LTS (Figure S1). As a first step, we assessed the distribution of PN, CL and Mes gene expression subtypes [20] across the expanded GT cohort. Classification into molecular subtypes demonstrated 10.7% PN, 42.8% CL and 38.9% Mes tumours (Figure S2). Further analysis of OS based on molecular subtypes showed no significant differences between subtypes (p = 0.4) (Figure S3).

### (Phospho-)Protein analysis of GBM samples using RPPA demonstrates heterogeneity in key signaling pathways in GBM

**Fig. 1:**
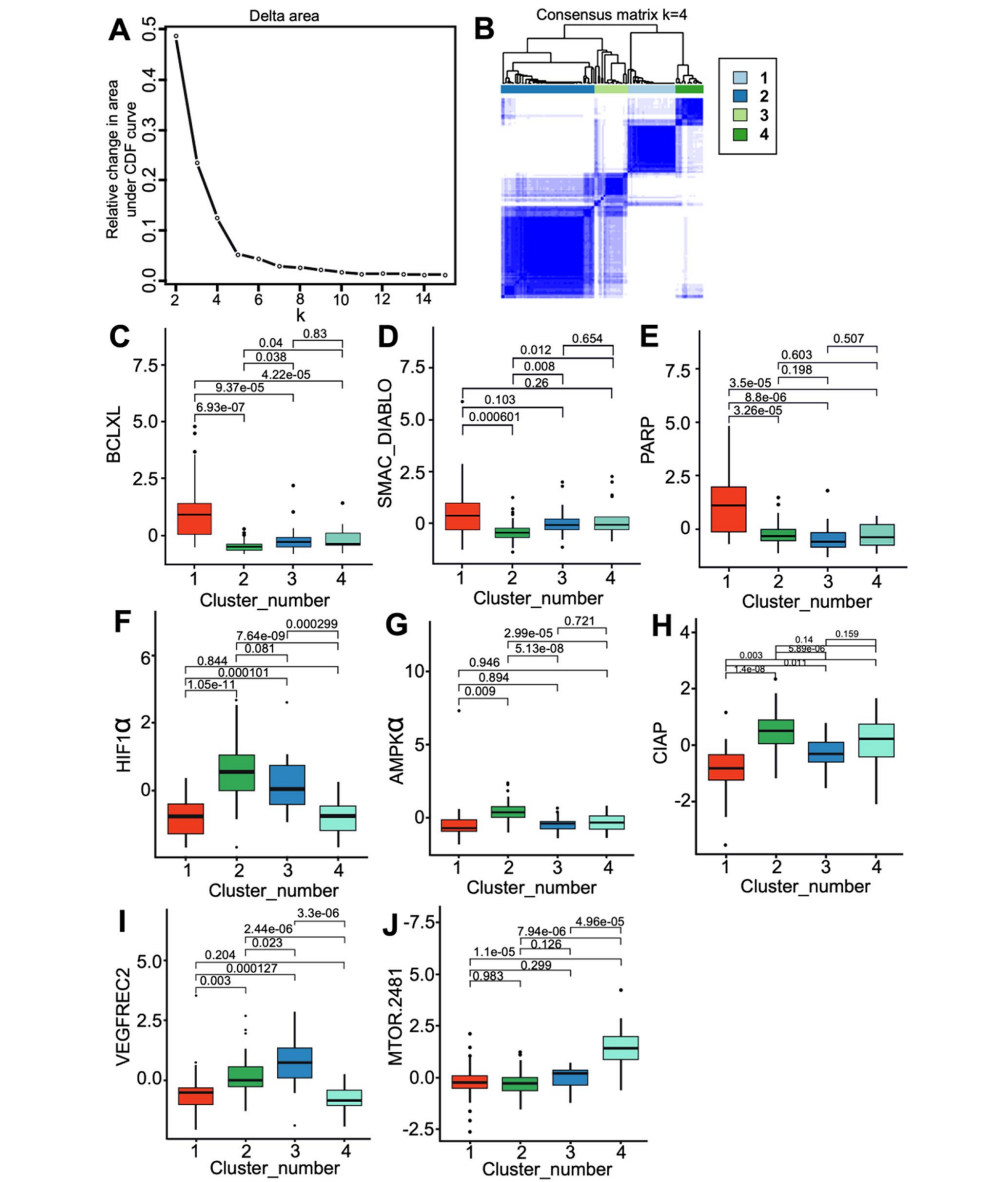
Identification of 4 (phospho-)protein clusters in GBM samples. (**A**) Elbow plot indicates the relative change in area under CDF curve vs. the k clusters. (**B**) Unsupervised CNF clustering for N = 126 samples indicates 4 distinct clusters. Boxplots reveal higher median expression of protein levels at cluster 1 for BCLXL (**C**), SMAC/DIABLO (**D**), and PARP (**E**); cluster 2 for HIF1α (**F**), AMPKα (**G**), and CIAP (**H**); cluster 3 for VEGFREC2 (**I**); and cluster 4 for mTOR(2481) (**J**). Likelihood-ratio t-test was used to calculate the significant difference between each cluster (ANOVA, Tukey HSD p-adjust. < 0.05)

RPPA analysis quantified signaling proteins in N =126 samples of the expanded GT cohort [21], which first underwent patient-to-patient clustering to identify potential (phospho)proteomic subtypes. 4 distinct clusters were identified: cluster 1–4, consisting of 30, 58, 21, and 17 samples respectively (Fig. 1 A and 1B**)**. Specifically, cluster 1 exhibited high expression of apoptotic signaling proteins BCLXL, SMAC/DIABLO (Fig. 1C and D) and BAX (Figure S4), as well as PARP (Fig. 1E), PDK1 and FAK (Figure S4). Next, cluster 2 had high levels of HIF1α, AMPKα, cIAP (Fig. 1F-H), cleaved Caspase-9, Caspase 9 (D1315 and D330), and APAF1 (Figure S5 A-D). Cluster 3 had significantly higher levels of VEGFR2 (Fig. 1I), while cluster 4 showed increased expression of mTOR (Fig. 1J). Pairwise t-test with ANOVA, Tukey HSD p-adjust. < 0.05 was applied to compare between clusters.

We repeated clustering to group proteins with similar expression across samples. Normalized protein expression and clinical parameters of all patients is demonstrated via heatmap (Figure S6). We next investigated how clinical factors affected protein levels. Patients of different sex, age, and GBM subtypes were evenly distributed across clusters. Notably, LTS patients were found in clusters 1, 2, and 3 (26.66%, 24.13%, 38.09%), while STS samples were mainly in clusters 2 and 3 (17.24%, 28.57%), but not in clusters 1 and 4.

**Fig. 2:**
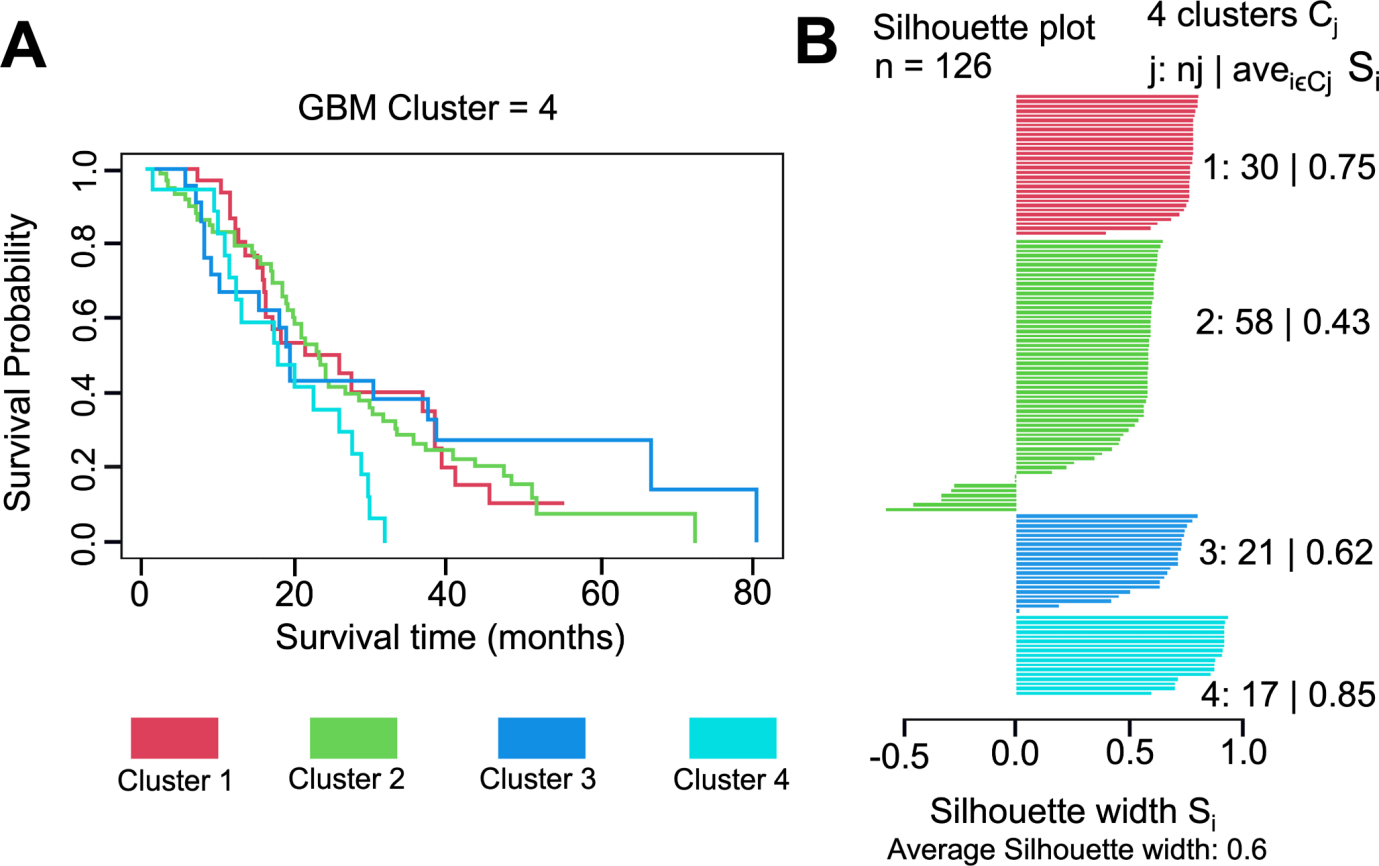
OS and silhouette plot for RPPA data across the GT cohort. (**A**) OS analysis plot showing the survival rate of all samples present in four different clusters. (**B**) Silhouette plot displayed a measure of how close each sample in one cluster is to sample in the neighbouring clusters. ((N = 30 (cluster 1), N = 58 (cluster 2), N = 21 (cluster 3) and N = 17 (cluster 4))

Cluster-specific survival was analysed using patients’ OS time in months (Fig. 2). Although cluster 4 had a trend towards shorter OS compared to clusters 1–3, no significant differences were found between clusters (pairwise t-test, p = 0.08) (Fig. 2A). Silhouette analysis indicated that clusters 1 and 4 were the most clearly defined clusters, with the highest separation (Fig. 2B). Cluster 1 had a silhouette coefficient of 0.75, and cluster 4 had a coefficient of 0.85, while clusters 2 and 3 had coefficients of 0.43 and 0.62, respectively.

### Elevated levels of GAB1 (Y627), SRC (Y527), BCL2 (S70) and RAF (S338) phosphoproteins associate with OS and are differentially expressed in LTS and STS

To investigate the association of individual proteins with OS, we fitted 72 univariate Cox regression models across the expanded-GT cohort (N = 126). Six proteins significantly correlated with OS [p27, GAB1 (Y627), SRC (Y527), BCLXL, BCL2 (S70), and RAF (S338)] (Table S1; Likelihood ratio p-value < 0.05). Nevertheless, no significantly correlated proteins were identified when adjusted for multiple comparisons.

**Fig. 3:**
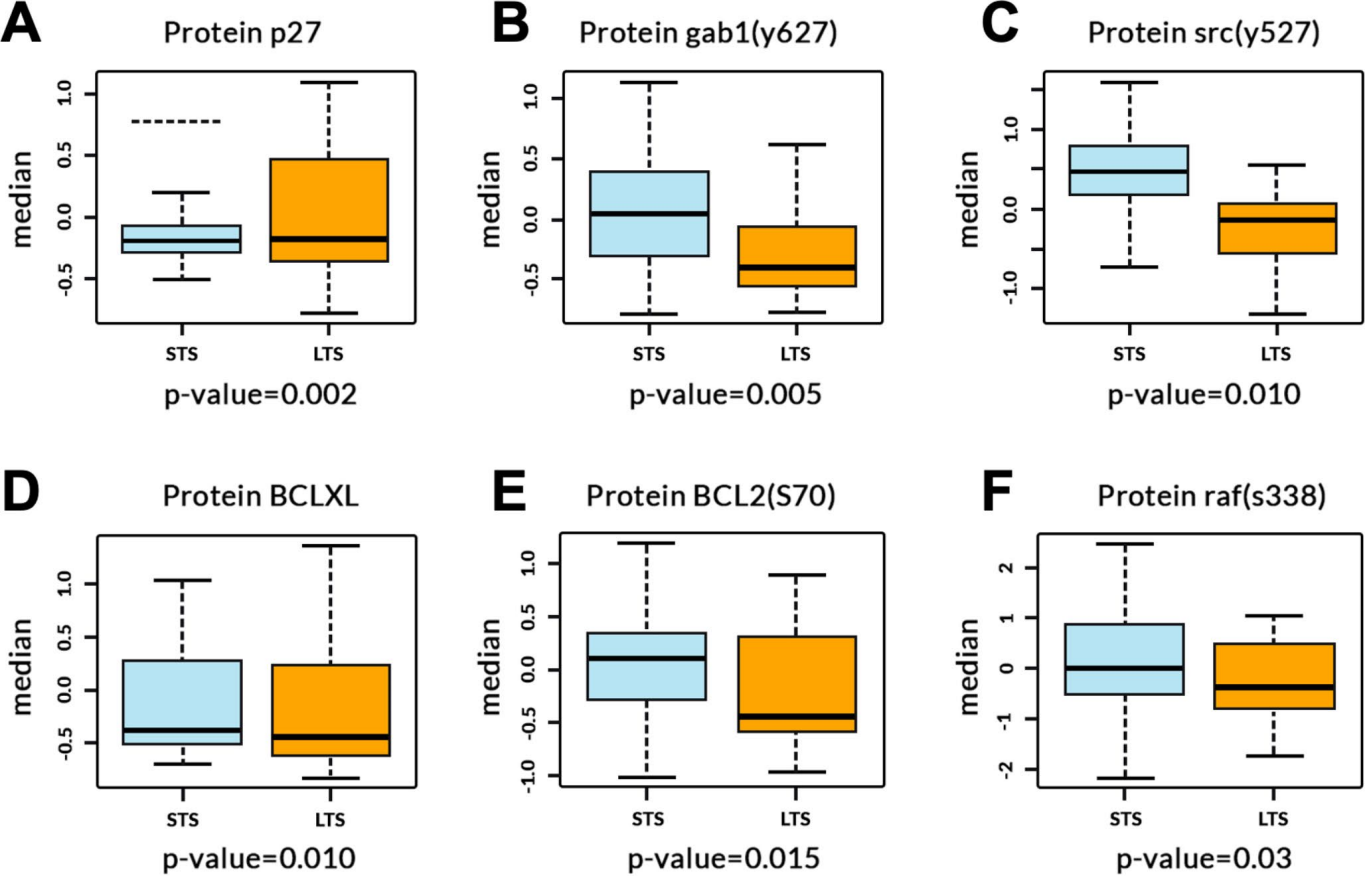
Proteins differentiating STS and LTS. Boxplots for proteins P27, GAB1 Y627, SRC Y527, BCLXL, BCL2 S70 and RAF S338 with p value for STS and LTS in GLIOTRAIN cohort. Likelihood ratio p-value < 0.05. N = 18(STS), N = 30(LTS)

As expected, we also identified significant differences in median levels of all six proteins (Table S1) between STS and LTS samples when analysed via pairwise t-test (Fig. 3A-F). Median levels of phosphorylated SRC (Y527) (p = 0.010; -0.15 and 0.49 median level in LTS and STS), GAB1 (p = 0.005; 0.45 and 0.01), BCL2 (p = 0.015; -0.49 and 0.12), RAF (p = 0.03; -0.5 and 0.01) and BCLXL (p = 0.010; -0.49 and − 0.46) expression were significantly lower in samples of patients with LTS compared to STS samples. In contrast, we found significantly greater median levels of P27 expression (p = 0.002) (-0.153 median level) in LTS samples compared to STS.

### Transcriptomic analysis of STS and LTS

**Fig. 4:**
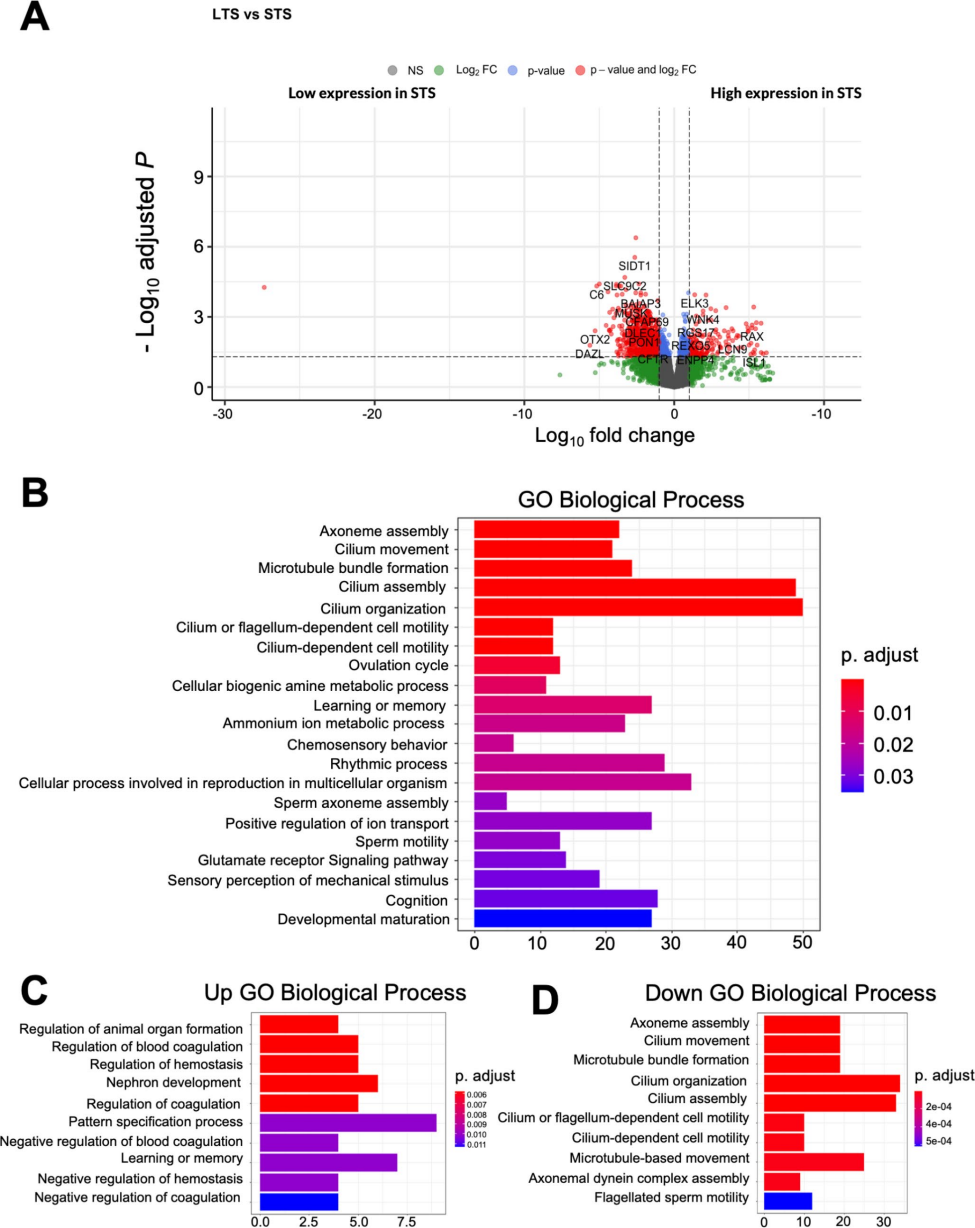
(**A**). Volcano plot showing DEGs for STS vs. LTS samples. (**B**) Overall, GO analysis for DEGs. (N = 1577). (**C**) GO annotations for up regulated genes enriched in STS samples. (**D**) GO annotations for down regulated genes enriched in LTS samples

We analyzed gene expression variations between STS and LTS patient tumors to create transcriptomic signatures to define survival groups. Our analysis identified N =1577 differentially expressed genes (DEGs) (N = 737 downregulated and N = 99 upregulated) altered between the two groups (Fig. 4A). Among these, Complement C6 (C6), Orthodenticle Homeobox 2 (OTX2), and Deleted in AZoospermia (DAZL) were the most differentially expressed down-regulated genes in LTS, while Retinal and Anterior Neural fold Homeobox (RAX) and Insulin gene enhancer protein ISL-1 (ISL1) were the most differentially expressed up-regulated genes in STS.

GO enrichment analysis was used to identify enriched pathways for DEGs in LTS and STS samples. GO Biological process (BP) terms were mainly associated with cilium gene ontologies such as cilium movement, microtubule bundle formation, cilium assembly, and cilium organization for extreme responders (p.adjust < 0.01) (Fig. 4B). Upregulated DEGs in STS were enriched with terms such as animal organ formation, regulation of blood coagulation, and regulation of homeostasis (p.adjust < 0.006), among other developmental terms (Fig. 4C). Conversely, downregulated DEGs in STS were highly enriched in microtubule bundle formation, cilium movement, organization and assembly, as well as cilium and flagellum dependent cell motility (p.adjust < 2e-04) (Fig. 4D).

### A cilium gene signature is prognostic within the GT cohort

**Fig. 5:**
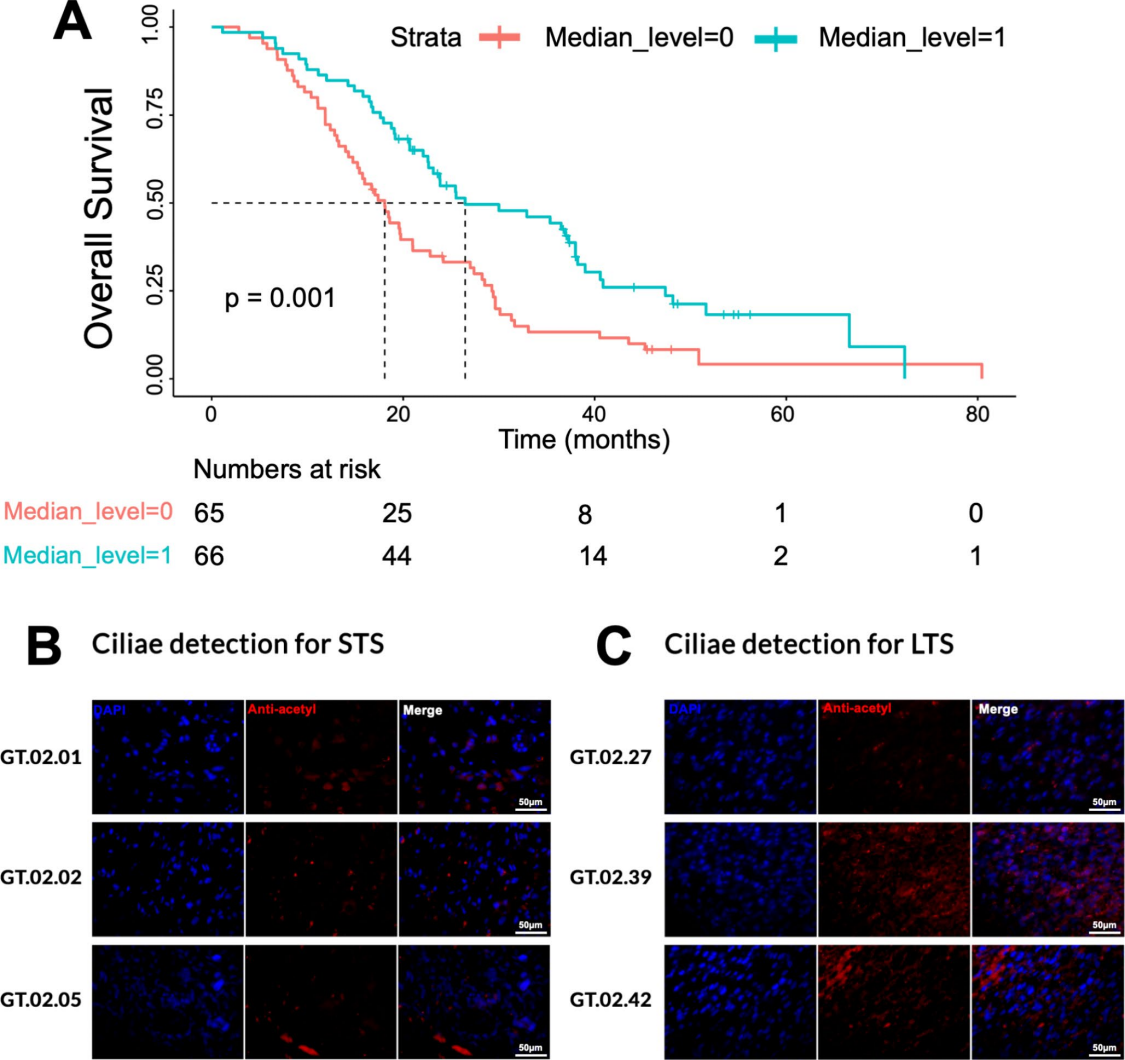
(**A**) OS analysis for the median expression for the cilium genes mapped from GO analysis. 44 cilium genes were mapped, and their median expression were taken for the GT dataset. The pink line demonstrates lower level of expression from the median value and the blue line shows higher level of expression from the calculated median value. Detection of ciliae in (**B**) STS and (**C**) LTS GBM samples using an anti-acetyl-alpha Tubulin antibody (red; middle panel). Nuclei were counterstained with DAPI (blue). Scale bar = 50 μm

As GO analysis revealed most genes related to cilium annotations, we next assessed the relationship between patient survival and cilium gene expression. This identified a total of N = 44 genes involved in cilium gene ontologies. Survival analysis based on these 44 genes within the-expanded GT cohort, revealed an improved OS in tumours with higher cilium gene expression (p < 0.001) (Fig. 5A).

Since cilium gene signatures appear to be a positive prognostic maker in the expanded GT cohort, we performed IHC to assess ciliae presence in a representative cohort of LTS patients. We analysed three STS samples (GT.02.01, GT.02.02, GT.02.05) (Fig. 5B) and three LTS samples (GT.02.27, GT.02.39, GT.02.42) (Fig. 5C) for ciliae expression using anti-acetyl-alpha Tubulin as marker of ciliae [19]. Indeed, two of the three LTS samples analysed showed a strong presence of ciliae, which was not observed in any of the STS tumour samples.

### Master Regulator analysis reveals signalling proteins and upregulated TFs in STS samples

We used a Master Regulator analysis to identify new targetable signaling pathways by examining 1577 DEGs and locating clusters of TF binding sites in upstream regulatory regions. We identified 263 TFs and enhancers targeted by them, and then used CMA to identify two modules containing 13 TFs regulating regions of our genes of interest [17]. Using the TRANSPATH® database [18], we reconstructed the signaling network, identifying 25 distinct master regulators and 13 TFs in our upregulated gene network in STS samples in comparison to LTS samples (Figure S7). These could be novel targets for inhibiting overactivated signaling pathways in GBM. Table S2 shows the lists of identified master regulators and their associations with pathways, such as integrin signaling and cell cycle regulation. We did not find any significantly enriched TFs in promoters of downregulated genes in the STS versus LTS comparison, in accordance with previous workflow publications [22].

## Discussion

The project aimed to discover new prognostic biomarkers and new therapeutic targets for GBM. To achieve this, we compared patients with favorable outcomes (LTS) to those with unfavorable outcomes (STS) using the internal GT cohort and RNA-seq transcriptomics and RPPA molecular datasets. The differences between the LTS and STS samples were analysed to identify potential biomarkers.

RPPA analysis on the entire GT cohort identified 4 clusters of (phospho)-proteins in GBM, revealing significant signaling heterogeneity. Although no significant difference in patients’ OS was observed, cluster 1 showed higher levels of BCLXL, BAX, PARP, PDK1, and FAK compared to the other clusters [23]. FAK and BAX were identified as potential targets for GBM treatment, while samples in cluster 1 with higher BCLXL levels could be treated with BH3 mimetics, and those with elevated SMAC/DIABLO levels could benefit from Smac inhibitor therapy [23–25].

Cluster 2 exhibits higher levels of cleaved Caspase 9 and Caspase 9, cIAP1, HIF1α, APAF1, and AMPKα suggesting an activation of the mitochondrial caspase pathway and a dysfunctional vasculature with hypoxia-sustaining microenvironments [26, 27]. Cluster 3 has elevated VEGFR2 protein levels indicating it is enriched with the Mes subtype and may be more amenable to anti-angiogenic therapy [28, 29]. Finally, cluster 4 has high phosphorylation of mTOR protein at Ser-2481 indicating higher M2 phenotype expression in TAMs, and mTOR suppression might reduce it [30].

We found 6 potential protein markers associated with OS in the merged GT cohort. Indeed, SRC(Y527) and RAF are associated with cell proliferation pathways [31, 32], and STS samples had higher median levels of these proteins, indicating their contribution to cancer tissue growth. Moreover, GAB1, which plays a significant role in cancer cell signaling pathways [33], had elevated levels in STS samples and could lead to reduced OS. BCL2 median protein levels were also higher in STS samples compared to LTS samples, and drugs such as venetoclax which selectively inhibits BCL2 may be considered [34].

Our study investigated transcriptomic differences between STS and LTS in GBM samples. The analysis revealed an enrichment of cilium-related GO annotations in LTS samples, indicating a potential role of cilia in improving OS in GBM. Overall, 44 genes with cilium-related GO terms were identified, and higher median expression of these genes was associated with better prognosis. The presence of cilia in LTS was verified through IHC. Previous work has identified defects in ciliogenesis in glioblastoma [35], and cilia have been identified on cells expressing Ki67 and cells associated with pseudopalisading necroses [36]. Our study suggests that cilia may serve as a new prognostic biomarker and potential therapeutic target in GBM, and further validation and functional studies are required to explore their role.

Further analysis found master regulators of upregulated genes in STS, which are related to important pro-oncogenic signaling pathways, including cell cycle, inflammation regulation, STAT signaling, EMT, and integrin cell signaling pathways [37]. The integrin family of transmembrane adhesion receptors plays a crucial role in cell interactions with the surroundings, including cytoskeleton organization, stimulation of cell proliferation, and rescue from programmed cell death [38, 39]. Specific integrins are upregulated in tumour cells and stromal cells in the tumour microenvironment, suggesting that targeting integrins could be an effective therapeutic strategy for GBM treatment [38, 40].

## Conclusion

In conclusion, our study identified four novel clusters of IDHwt GBM based on a (phospho)proteomic analysis with potential for patient stratification. In addition, we provide evidence that contrasting patients with particularly favorable outcomes (LTS) to patients with unfavorable outcomes (STS) and subsequent (phospho)proteomic, transcriptomic and master regulator analysis allows for the identification of new potential prognostic protein biomarkers and therapeutic targets. Transcriptomic analysis has moreover suggested a novel association of cilium genes with survival of GBM patients. Future experimental and clinical validation of our key findings is required.

## Electronic supplementary material

Below is the link to the electronic supplementary material.

Supplementary Material 1

## Data Availability

The datasets generated during and/or analysed during the current study are available from the corresponding author on reasonable request.
