# Supplementary material for: Comparative analysis of deeply phenotyped GBM cohorts of ‘short-term’ and ‘long-term’ survivors
Source: J Neurooncol. 2023 May 26;163(2):327–38. doi: 10.1007/s11060-023-04341-3 (PMC10322749; doi:10.1007/s11060-023-04341-3)
Supplement: Supplementary file 1 — Supplementary Material 1 [file 11060_2023_4341_MOESM1_ESM.docx]

### Comparative analysis of deeply phenotyped GBM cohorts of ‘short-term’ and ‘long-term’ survivors

**Archita Biswas1, Manuela Salvucci1, Kate Connor1, Heiko Düssmann1, Steven Carberry1, Michael Fichtner1, Ellen King1, Brona Murphy1, A.C O’Farrell1, Jane Cryan2, Alan Beausang2, Josephine Heffernan2, Mattia Cremona3, Bryan T. Hennessy3, James Clerkin1,4, Kieron J. Sweeney4, Steve MacNally4, F Brett4, P O’Halloran4, Orna Bacon1, Simon Furney1, Maite Verreault5, Emie Quissac5, Franck Bielle5, Mohammed Ahmed5, Ahmed Idbaih5, Sieger Leenstra6, Ioannis Ntafoulis6, Federica Fabro6, Martine Lamfers6, Anna Golebiewska7, Frank Hertel7 and 11**, **Simone P Niclou7 and 11, Romain Tching Chi Yen8, Andreas Kremer8, Gonca Dilcan9, Francesca Lodi9, Ingrid Arijs9, Diether**

**Lambrechts9, Manasa K P10, Alexander Kel10, Annette T. Byrne1, Jochen H.M. Prehn1***

1 Department of Physiology and Medical Physics and Centre for Systems Medicine, Royal College of Surgeons in Ireland, 123 St Stephen's Green, Dublin 2, D02 YN77, Ireland

2 Department of Neuropathology, Beaumont Hospital, Dublin 9, Ireland

3.Department of Medicine, Royal College of Surgeons in Ireland and Beaumont Hospital, Dublin 9, Ireland

4Department of Neurosurgery, Beaumont Hospital, Dublin 9, Ireland

5 Sorbonne Université, AP-HP, Institut du Cerveau - Paris Brain Institute - ICM, Inserm, CNRS, Hôpitaux Universitaires La Pitié Salpêtrière - Charles Foix, DMU Neurosciences, Service de Neurologie 2-Mazarin, F-75013, Paris, France

6Dept of Neurosurgery Brain Tumor Center, Erasmus University Medical Center, Wytemaweg 80, 3015 CN Rotterdam, The Netherlands

7NORLUX Neuro-Oncology laboratory, Department of Cancer Research, Luxembourg Institute of Health, 6A, Rue Nicolas- Ernest Barblé, L-1210 Luxembourg

8Information Technology for Translational Medicine, 27, Rue Henri Koch - House of BioHealth, L-4354 Esch-sur-Alzette, G.D, Luxembourg

9 VIB-KU Leuven Center for Brain & Disease Research, Onderwijs en Navorsing 5, Herestraat, 49, 3000 Leuven, Belgium

10geneXplain GmbH, Am Exer 19b, 38302 Wolfenbüttel, Germany

11Faculty of Sciences, Technology and Medicine, University of Luxembourg, L-4365 Esch-sur-Alzette, Luxembourg

*Corresponding author:

Prof Jochen H. M. Prehn

Department of Physiology and Medical Physics RCSI Centre for Systems Medicine

RCSI University of Medicine and Health Sciences 123 St. Stephen’s Green

Dublin D02 YN77, Ireland Phone: +353 1 402 2255

Email: [prehn@rcsi.ie](mailto:prehn@rcsi.ie)


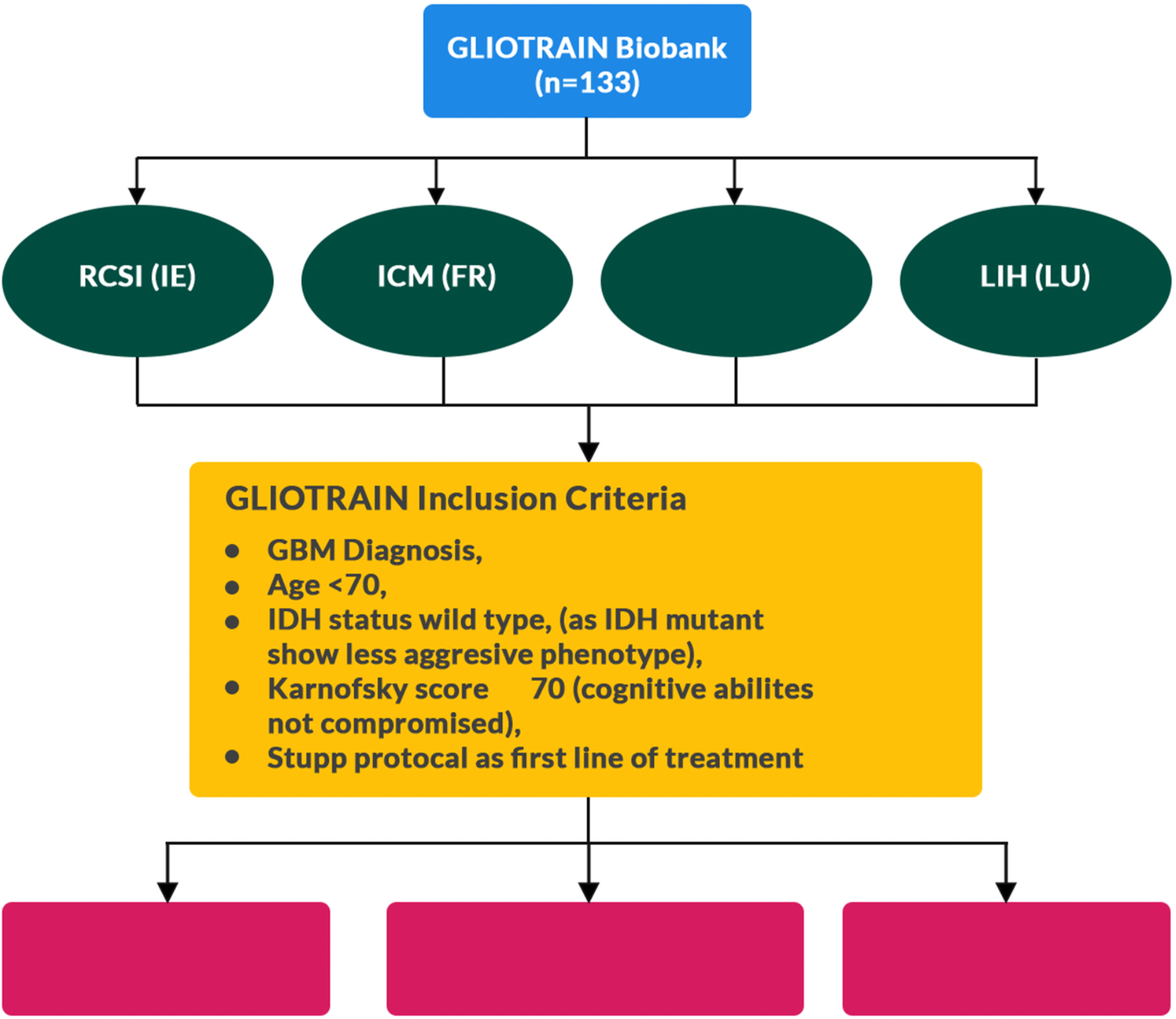

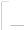

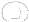

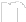

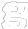

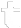

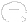

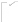

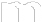

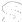

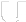

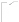

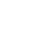

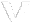

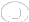

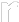

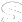

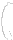

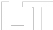

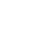

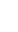

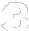

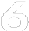

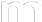

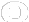

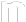

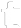

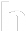

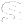

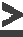

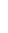

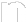

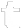

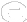

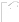

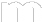

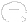

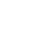

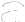

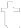

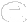

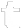

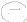

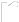

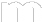

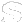

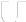

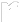

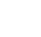

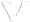

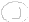

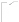

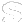

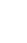

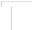

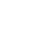

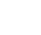

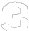

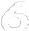

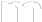

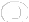

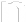

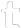

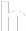

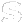

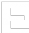

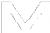

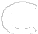

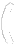

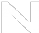

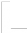

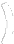

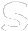

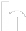

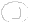

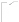

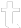

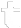

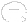

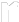

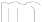

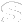

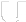

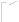

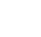

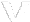

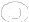

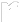

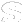

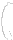

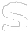

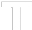

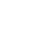

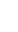

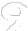

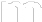

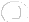

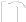

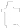

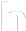


*Figure S1: Sample collection criteria of expanded GT cohort samples and stratification into short, intermediate and long term survivors based on extent of survival (months)*

*Figure S2: Pie Chart depicting the distribution of molecular subtypes for expanded-GT cohort*

Overall survival

*Figure S3:Overall survival analysis for expanded-GT cohort based on GBM subtypes*

Strata

Subtype=Classical

Subtype=Mesenchymal

Subtype=Proneural

1.00

0.75

0.50

0.25

p = 0.4

0.00

0

Number at risk

56

51

14

20

80

39

18

5

40

Time [months]

8

12

2

60

1

1

1

1

0

0

# A

7.5

5.0

2.5

0.0

1 2 3 4

0.009

1.08e-06

0.931

4.65e-07

0.024

0.002

**B**

6 0.002

0.000125

3

PDK1

0

1 2 3 4

0.954

0.84

0.946

0.000462

BAX

1 2 3 4 1 2 3 4

Cluster_number Cluster_number

**C** 1 2 3 4

0.052

15 0.002

0.015

0.008

0.001

10 0.000508

FAK

5

0

1 2 3 4

Cluster_number

*Figure S4: Boxplot showing higher median expression of protein levels at cluster 1. A) BAX B) PDK1 C) FAK*

# A

1

2

3

4

**B**

1

2

3

4

0.953

0.031 0.266

3.61e-07

0.256

0.951

1.77e-05

1.75e-05

2.87e-05

0.901

6

0.738

0.000499

3

0

-3

1

Cluster_number

2

3

4

1

Cluster_number

2

3

4

1

2

3

4

**D**

7.5

1

2

3

4

0.02

7.91e-07

0.000583

0.421

0.012

0.004

3.2e-13

0.116

0.283

5.33e-07

5.0

3.73e-08

3.71e-15

2.5

0.0

12

CASP9(D1315)

8

CASP9(D330)

4

0

4

**C**

2

0

CASP9

APAF1

-2

-4

1 2 3 4

1 2 3 4

## Cluster_number Cluster_number

*Figure S5: Boxplot showing higher median expression of protein levels at cluster 2. A) CASP9(D1315)*

*B) CASP9(D330) C) CASP9 D) APAF1*

*Figure S6: Complex heatmap showing normalized protein expression in x axis versus patient’s clinical parameters such as: sex, centres, age at diagnosis, MGMT status, OS event and month, survivor’s type, subtypes of GBM, and lastly the cluster numbers.in y axis.*

|  | **Hazard ratio**  **(HR)** | **CI**  **(lower .95)** | **CI**  **(upper .95)** | **p-value** |
| --- | --- | --- | --- | --- |
| **p27** | 1.79 | 1.24 | 2.58 | 0.002 |
| **Gab1 (y627)** | 1.57 | 1.18 | 2.08 | 0.005 |
| **Src (y527)** | 1.33 | 1.06 | 1.66 | 0.010 |
| **BCLXL** | 0.78 | 0.63 | 0.97 | 0.010 |
| **BCL2 (S70)** | 0.78 | 0.64 | 0.95 | 0.015 |
| **Raf (s338)** | 0.78 | 0.62 | 0.99 | 0.03 |

*Table S1: Univariate cox regression analysis identifies proteins significantly associated with overall survival.*

*Figure S7: Master Regulator analysis for the differentially expressed up regulated genes enriched in STS samples. The gene regulatory network for upregulated genes is displayed (Log2FC > 0 and padj < 0.05). Orange outside fillings suggest master regulators that are up regulated in our dataset. Green blocks indicate intermediate molecules regulating the network. Blue blocks represent molecules which directly affect the Master regulators. Purple blocks indicate transcription factors that are present in the network.*

### Master regulators

**Gene description Gene symbol**

| **PDGFRalpha(h)** | platelet derived growth factor receptor alpha | PDGFRA |
| --- | --- | --- |
| **integrins** | integrin subunit alpha 1, integrin subunit alpha 2b, integrin subunit alpha 3, integrin subunit alpha 4, integrin subunit alpha 5, integrin subunit alpha 6, integrin subunit alpha 8, integrin subunit alpha 9, integrin subunit alpha L, integrin subunit alpha V, integrin subunit beta 1, integrin subunit beta 2, integrin subunit beta 3, integrin subunit beta 4, integrin subunit beta 5, integrin subunit beta 6 | ITGA1, ITGA2B, ITGA3, ITGA4, ITGA5, ITGA6, ITGA8, ITGA9, ITGAL, ITGAV, ITGB1, ITGB2, ITGB3, ITGB4, ITGB5, ITGB6 |
| **BMP2A(h)** | bone morphogenetic protein 2 | BMP2 |
| **IRAK-2(h)** | interleukin 1 receptor associated kinase 2 | IRAK2 |
| **IL-8(h):CXCR1(h):G-alpha-**  **i2(h)** | C-X-C motif chemokine ligand 8, C-X-C motif chemokine receptor 1, G protein subunit alpha i2 | CXCL8, CXCR1, GNAI2 |
| **plk1(h)** | polo like kinase 1 | PLK1 |
| **plk1(h)(Silke and Meier)** | polo like kinase 1 | PLK1 |
| **Cdc25B(h)** | cell division cycle 25B | CDC25B |
| **Cdc25B(h)(Kalya et al.)** | cell division cycle 25B | CDC25B |
| **Cdc25B-isoform3(h)** | cell division cycle 25B | CDC25B |
| **gamma-secretase(h)** | aph-1 homolog A, gamma-secretase subunit, nicastrin, presenilin 1, presenilin enhancer, gamma-secretase subunit | APH1A, NCSTN, PSEN1, PSENEN |
| **LPS:lbp:CD14:TLR4:MD- 2:TIRAP:IRAK-2** | CD14 molecule, TIR domain containing adaptor protein, interleukin 1 receptor associated kinase 2, lipopolysaccharide binding protein, lymphocyte antigen 96, toll like receptor 4 | CD14, IRAK2, LBP, LY96, TIRAP, TLR4 |
| **IL-1beta-p17:IL-1RI:IL-**  **1RAcP:MyD88:tollip:IRAK- 1{pS376}{pT387}:IRAK-**  **4:IRAK-2** | MYD88 innate immune signal transduction adaptor, interleukin 1 beta, interleukin 1 receptor accessory protein, interleukin 1 receptor associated kinase 1, interleukin 1 receptor associated kinase 2,  interleukin 1 receptor associated kinase 4, interleukin 1 receptor type 1, toll interacting protein | IL1B, IL1R1, IL1RAP, IRAK1, IRAK2, IRAK4, MYD88, TOLLIP |
| **Cdc20(h)** | cell division cycle 20 | CDC20 |
| **cyclosome(h):Cdc20(h)** | anaphase promoting complex subunit 1, anaphase promoting complex subunit 10, anaphase promoting complex subunit 11, anaphase promoting complex subunit 2, anaphase promoting complex subunit 4, anaphase promoting complex subunit 5, anaphase promoting complex subunit 7, cell division cycle 16, cell division cycle 20, cell division cycle 23, cell division cycle 26, cell division cycle 27 | ANAPC1, ANAPC10, ANAPC11, ANAPC2, ANAPC4, ANAPC5, ANAPC7, CDC16, CDC20, CDC23, CDC26, CDC27 |
| **Nek2A(h)** | NIMA related kinase 2 | NEK2 |
| **Nek2A(h){p}** | NIMA related kinase 2 | NEK2 |
| **plk1(h)** | polo like kinase 1 | PLK1 |
| **cyclosome(h):Cdc20(h){ub} n** | anaphase promoting complex subunit 1, anaphase promoting complex subunit 10, anaphase promoting complex subunit 11, anaphase promoting complex subunit 2, anaphase promoting complex subunit 4, anaphase promoting complex subunit 5, anaphase promoting complex subunit 7, cell division cycle 16, cell division cycle 20, cell division cycle 23, cell division cycle 26, cell division cycle 27 | ANAPC1, ANAPC10, ANAPC11, ANAPC2, ANAPC4, ANAPC5, ANAPC7, CDC16, CDC20, CDC23, CDC26, CDC27 |
| **Dkk-1(h)** | dickkopf WNT signaling pathway inhibitor 1 | DKK1 |
| **usp13-isoform1(h)** | ubiquitin specific peptidase 13 | USP13 |
| **usp13(h)** | ubiquitin specific peptidase 13 | USP13 |
| **RPTPepsilon(h)** | protein tyrosine phosphatase receptor type E | PTPRE |
| **CUL4A(h):DDB1(h):DTL(h)** | cullin 4A, damage specific DNA binding protein 1, denticleless E3 ubiquitin protein ligase homolog | CUL4A, DDB1, DTL |
| **Cdk6(h):cyclinD3-**  **isoform1(h)** | cyclin D3, cyclin dependent kinase 6 | CCND3, CDK6 |

*Table S2: 25 Identified master regulators for STS upregulated genes.*
